# Reduced mortality associated with pulmonary embolism response team consultation for intermediate and high-risk pulmonary embolism: a retrospective cohort study

**DOI:** 10.1186/s12959-024-00605-8

**Published:** 2024-04-19

**Authors:** Tiffany A. Gardner, Alexandra Fuher, August Longino, Eric M. Sink, James Jurica, Bryan Park, Jonathan Lindquist, Todd M. Bull, Peter Hountras

**Affiliations:** 1 Pulmonary and Critical Care Fellowship Program, Massachusetts General Hospital & Beth Israel Deaconess Medical Center, Boston, MA 02114 USA; 2 Internal Medicine Residency Program, Department of Medicine, University of Colorado Anschutz Medical Campus, Aurora, CO USA; 3 Division of Pulmonary Sciences & Critical Care, Pulmonary Vascular Disease Center, Department of Medicine, University of Colorado Anschutz Medical Campus, Aurora, CO USA; 4 Division of Vascular and Interventional Radiology, Department of Radiology, University of Colorado Anschutz Medical Campus, Aurora, CO USA

## Abstract

**Background:**

The management of acute pulmonary embolism (PE) has become increasingly complex with the expansion of advanced therapeutic options, resulting in the development and widespread adoption of multidisciplinary Pulmonary Embolism Response Teams (PERTs). Much of the literature evaluating the impact of PERTs has been limited by pre- postimplementation study design, leading to confounding by changes in global practice patterns over time, and has yielded mixed results. To address this ambiguity, we conducted a retrospective cohort study to evaluate the impact of the distinct exposures of PERT availability and direct PERT consultation.

**Methods:**

At a single tertiary center, we conducted propensity-matched analyses of hospitalized patients with intermediate or high-risk PE. To assess the impact of PERT availability, we evaluated the changes in 30-day mortality, hospital length of stay (HLOS), time to therapeutic anticoagulation (TAC), in-hospital bleeding complications, and use of advanced therapies between the two years preceding and following PERT implementation. To evaluate the impact of direct PERT consultation, we conducted the same analyses in the post-PERT era, comparing patients who did and did not receive PERT consultation.

**Results:**

Six hundred eighty four patients were included, of which 315 were pre-PERT patients. Of the 367 postPERT patients, 201 received PERT consultation. For patients who received PERT consultation, we observed a significant reduction in 30-day mortality (5% vs 20%, OR 0.38, *p* = 0.0024), HLOS.

(-5.4 days, *p* < 0.001), TAC (-0.25 h, *p* = 0.041), and in-hospital bleeding (OR 0.28, *p* = 0.011).

These differences were not observed evaluating the impact of PERT presence in pre-vs postimplementation eras.

**Conclusions:**

We observed a significant reduction in 30-day mortality, hospital LOS, TAC, and in-hospital bleeding complications for patients who received PERT consultation without an observed difference in these metrics when comparing the pre- vs post-implementation eras. This suggests the benefits stem from direct PERT involvement rather than the mere existence of PERT. Our data supports that PERT consultation may provide benefit to patients with acute intermediate or high-risk PE and can be achieved without a concomitant increase in advanced therapies.

**Supplementary Information:**

The online version contains supplementary material available at 10.1186/s12959-024-00605-8.

## Background

Venous thromboembolism (VTE), including pulmonary embolism (PE) and deep venous thrombosis, remains a leading cause of morbidity and mortality worldwide [1]. There are an estimated 900,000 cases of VTE in the United States annually, with approximately one third of.

cases resulting in long term complications and 11% of cases resulting in patient mortality [1, 2].

Appropriate treatment of patients with PE requires rapid diagnosis and risk-stratification at the time of presentation based on blood pressure and imaging or biomarker evidence of right heart strain [3, 4]. There is guideline consensus regarding the management of patients with both low and high-risk PE; however, uncertainty persists regarding the management of patients with intermediate-risk disease [2, 4, 5]. These patients represent 20–45% of all patients with PE and are at elevated risk of decompensation [6]. In recent years, new techniques have been developed to aid in the management of patients presenting with hemodynamically significant PE. These options include catheter directed thrombolysis (CDT), percutaneous thrombectomy, mechanical circulatory support, Extracorporeal Membrane Oxygenation (ECMO), and others [7]. Treatment of patients with intermediate-risk PE remains challenging due to variability in patient presentation, a growing number of possible procedural interventions, and an evolving body of evidence for the efficacy and risk of available treatment options.

In response to the growing complexity of intermediate-risk PE management, the first multidisciplinary pulmonary embolism response team (PERT) was established more than a decade ago to engage in nuanced discussion of patient-specific factors and rapidly identify and implement the most appropriate treatment. Since that time, there has since been widespread adoption of the PERT model, including integration into recent consensus-based guidelines [4, 8]. Common members of PERTs include providers trained in pulmonary and critical care medicine, cardiology, hematology, emergency medicine, interventional radiology, cardiothoracic surgery, pharmacy, and other stakeholders, though the structure and composition of PERTs vary widely between institutions. Prior evaluations of PERTs at single centers and by a multi-center PERT consortium have demonstrated mixed findings regarding their benefit, with some retrospective evidence for benefit among patients with intermediate and high-risk PE [9]. Many prior studies have compared patient outcomes during periods before and after PERT initiation – analyses that may be confounded by global practice changes [10, 11].

Our study is among the first to evaluate not only the clinical impact of the existence of a PERT at the time of PE diagnosis (“PERT presence” or “PP”), but also direct consultation of PERT (“PERT consultation” or “PC”), for patients with intermediate and high-risk PE at a large quaternary care center.

## Methods

### Study Design

**Fig. 1:**
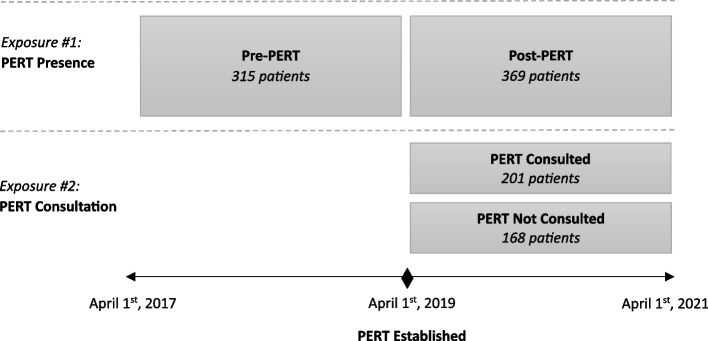
Study design

We adhered to STROBE reporting guidelines [12]. We performed a retrospective, single-center cohort study at a large quaternary care center. We included patients who were admitted with a diagnosis of intermediate or high-risk PE in the two years preceding and following PERT implementation, between April 2017 and April 2021. Patients were excluded if diagnosed prior to transfer from a referring facility. The aim of this study was to evaluate the clinical impact of PERT presence and PERT consultation (Fig. 1):

- *PERT Presence:* To evaluate the impact of establishing a PERT, we compared clinical outcomes in the two years preceding and following PERT implementation: from April 2017 to March 2019, as compared to April 2019 to April 2021.
- *PERT Consultation:* To evaluate the impact of PERT consultation, we compared clinical outcomes in patients who received PERT consultation (PC) and those who did not (NPC) in the two years following PERT implementation (post-implementation era): from April 2019 to April 2021.

We queried our electronic medical record for all encounters during the specified study period for patients with a diagnosis of acute PE. Patient encounters were manually reviewed to ensure that PE classification met criteria as intermediate or high-risk. Extracted data included patient.

demographics, vital signs, Pulmonary Embolism Severity Index (PESI) [3, 4]at the time of PE diagnosis, laboratory and imaging evidence of right heart strain, PERT consultation, interventions, among others. Outcomes of interest included 30-day mortality, hospital length of stay (HLOS), time to therapeutic anticoagulation (TAC), and in-hospital bleeding. The dataextraction protocol and kappa-statistics for consistency between researchers is presented in Appendix 1. Data were collected and stored in a secure, HIPAA-compliant online database (REDCap). Frequency and type of procedural intervention and systemic thrombolysis were also assessed.

### Statistical analysis

Summary statistics were tabulated and stratified by PERT availability and consultation, using Wilcoxon-Rank-Sum test and Pearson’s Chi-squared or Fisher’s exact tests for continuous and categorical variables, respectively.

We conducted a propensity-score matched analysis [13] to estimate the average marginal effect of PERT consultation on a variety of outcomes including 30-day mortality, HLOS, in-and hospital bleeding complications. We accounted for confounding by including covariates of age, sex, PESI score, use of home anticoagulation, history of COVID-19, congestive heart failure, chronic obstructive pulmonary disease, cirrhosis, and cancer. Covariates were selected a-priori based on clinical relevance. We used full matching on the propensity score, which yielded adequate balance, as indicated in Appendix 1. The propensity score was estimated using a probit regression of the treatment on the covariates, which yielded adequate balance, with absolute standardized mean differences and Kolmogorov–Smirnov Statistics for all covariates of < 0.2.

Full matching uses all treated and all control units, so no units were discarded by the matching.

To estimate the treatment effect and its standard error, we used matched multivariable regression models to determine the impact of PERT presence and consultation on the outcomes listed above. We used linear models for continuous dependent variables and logistic regression for binary dependent variables using the treatment, covariates, and their interaction as predictors and included the full matching weights in the estimation. A cluster-robust variance was used to estimate its standard error with matching stratum membership as the clustering variable.

Propensity-matching analyses were conducted using the MatchIt package [14] for R computing software version 4.2.1 (2022–06-23).

## Results

### PERT Presence

Six hundred eighty four patients were included, of which 315 were pre-PERT patients. Pre- and post-PERT patients were similar in age, sex, PE classification, and PESI scores (Table 1). PrePERT patients were more likely to have active cancer (*p* < 0.05) or be prescribed anticoagulation prior to admission (*p* < 0.05) and less likely to have a history of COVID-19 pneumonia (*p* < 0.05).

**Table 1:** Patient characteristics, clinical covariates, and clinical findings stratified by PERT presence and PERT consultation

	**PERT PRESENT**			**PERT CONSULTED**		
	**Pre-PERT:** *n* = *319 patients*^*1*^	**Post-PERT:** *n* = *367 patients*^*1*^	***p*-value**^2^	**PERT NOT** **CONSULTED:** *n* = *166 patients*^*1*^	**PERT** **CONSULTED:** *n* = *201 patients*^*1*^	***p*-value**^**2**^
**Patient demographics**						
Age (Years)	62 (52, 73)	64 (53, 73)	0.6	65 (53, 74)	63 (53, 72)	0.4
Sex			0.4			0.7
Male	175 (54.9%)	212 (58%)		98 (59.0%)	114 (56.7%)	
Female	144 (45.1%)	155 (42%)		68 (41.0%)	87 (43.3%)	
**Clinical covariates**						
COPD	37 (11.6%)	41 (11.2%)	> 0.9	11 (6.6%)	30 (14.9%)	0.012*
Congestive heart failure	51 (16.0%)	56 (15.3%)	0.8	33 (19.9%)	23 (11.4%)	0.025*
ILD	8 (2.5%)	11 (3.0%)	0.7	8 (4.8%)	3 (1.5%)	0.072
Pulmonary HTN	28 (8.8%)	30 (8.2%)	0.8	11 (6.6%)	19 (9.5%)	0.3
Active Cancer	104 (32.6%)	83 (22.6%)	0.003*	48 (28.9%)	35 (17.4%)	0.009*
History of Venous Thromboembolism	66 (20.7%)	56 (15.3%)	0.064	19 (11.4%)	37 (18.4%)	0.065
Cirrhosis	4 (1.3%)	7 (1.9%)	0.5	6 (3.6%)	1 (0.5%)	0.049*
History of GI Bleed	12 (3.8%)	15 (4.1%)	0.8	7 (4.2%)	8 (4.0%)	> 0.9
History of Thrombophilia	8 (2.5%)	9 (2.5%)	> 0.9	1 (0.6%)	8 (4.0%)	0.044*
Autoimmune Disease	47 (14.7%)	48 (13.1%)	0.5	25 (15.1%)	23 (11.4%)	0.3
History of COVID-19	0 (0%)	44 (12%)	< 0.001*	24 (14%)	20 (10.0%)	0.3
Home anticoagulation	25 (7.8%)	14 (3.8%)	0.023*	8 (4.8%)	6 (3.0%)	0.4
Apixaban	6 (24.0%)	3 (21.4%)		2 (25.0%)	1 (16.7%)	
Dabigatran	1 (4.0%)	0 (0.0%)		0 (0.0%)	0 (0.0%)	
Dalteparin	2 (8.0%)	1 (0.0%)		1 (0.0%)	0 (0.0%)	
Edoxaban	0 (0.0%)	2 (7.1%)		2 (12.5%) 0	1 (0.0%)	
Enoxaparin	4 (16.0%)	1 (7.1%)		(0.0%)	2 (16.7%)	
Rivaroxaban	5 (20.0%)	3 (21.4%)		1 (12.5%)	3 (33.3%)	
Warfarin	7 (28.0%)	6 (42.9%)		4 (50.0%)	2 (33.3%)	
Active Bleed at Diagnosis	19 (6.0%)	21 (5.7%)	0.9	15 (9.0%)	6 (3.0%)	0.013*
Presence of PE contributed to admission	226 (70.8*%)	270 (73.6%)	0.5	96 (57%)	175 (87%)	** < 0.001**
**Clinical findings**						
Troponin-I	0.10 (0.02, 0.44)	0.08 (0.02, 0.3)	0.3	0.07 (0.02, 0.22)	0.10 (0.03, 0.36)	0.11
BNP	228 (82, 592)	201 (71, 559)	0.3	200 (74, 462)	201 (70, 563)	0.9
Echocardiographic Evidence of RH Strain			0.2			< 0.001*
Yes	227 (71.2%)	263 (71.7%)		97 (58.4%)	166 (82.6%)	
No	54 (16.9%)	74 (20.2%)		40 (24.1%)	34 (16.9%)	
Not Obtained	38 (11.9%)	30 (8.2%)		29 (17.5%)	1 (0.5%)	
CT Evidence of RH Strain			0.4			< 0.001*
Yes	193 (60.5%)	221 (60.2%)		76 (46%)	145 (72.1%)	
No	111 (34.8%)	136 (37.1%)		85 (51%)	51 (25.4%)	
Not Obtained	15 (4.7%)	10 (2.7)		5 (3.0%)	5 (2.5%)	
Full Risk Stratification Obtained (troponin or BNP, plus CT or TTE)	196 (61.4%)	277 (75.5%)	< 0.001*	89 (53.6%)	188 (93.5%)	< 0.001*
PE Classification			0.2			0.004*
High-Risk	36 (13.2%)	31 (9.1%)		12 (8.5%)	19 (9.6%)	
Intermediate-High Risk	151 (55.3%)	188 (55%)		65 (46%)	123 (62%)	
Intermediate-Low Risk	86 (31.5%)	121 (36%)		65 (46%)	56 (28%)	
Not Risk Stratified	46 (14.4%)	27 (7.4%)		24 (14.5%)	3 (1.5%)	
PESI	106 (83,134)	105 (82, 138)	0.7	110 (85, 138)	104 (80, 138)	0.6

^1^Median (IQR), *n* (%)

^2^Wilcoxon rank sum test, Pearson’s Chi-squared test, Fisher’s exact test

Patients in the post-implementation era were more likely to have full risk stratification obtained (assessment of PE risk using both imaging and biomarker data) than patients in the preimplementation era (61.4% vs 75.5%, *p* < 0.001). There were significantly fewer procedures following PERT implementation (20.8% vs 11.4%, *p* = 0.01, Table 2). Post-PERT patients were less likely to undergo catheter directed lysis (4.7% vs 0.5%, *p* < 0.001), as well as a trend towards being less likely to undergo all catheter-based procedures (6.3% vs 4.6%, *p* = 0.67). Post-PERT patients were also less likely to undergo IVC filter placement (13.2% vs 6.5%, *p* = 0.003). No patients in the pre- or post-implementation eras were treated with ECMO. There was also a nonsignificant trend towards an increase in thromboaspiration procedures (1.6% vs 4.1%. *p* = 0.05). A higher percentage of post-PERT patients received enoxaparin as an initial anticoagulant (30% vs 18%, *p* < 0.05). There were no significant differences in 30-day mortality, HLOS, TAC, or inhospital bleeding complications after controlling for demographics and common comorbidities between the pre- and post-PERT eras (Table 3).

**Table 2:** Interventions and patient outcomes stratified by PERT presence and PERT consultation

	**PERT PRESENT**			**PERT CONSULTED**		
**Interventions and Patient Outcomes**	**Pre-PERT:** *n* = *317 patients*^*1*^	**Post-PERT:** *n* = *369 patients*^*1*^	***p*-value**^2^	**PERT NOT** **CONSULTED:** *n* = *168 patients*^*1*^	**PERT** **CONSULTED:** *n* = *201 patients*^*1*^	***p*-value**^2^
Systemic Anticoagulant	308 (96.6%)	355 (96.7%)	0.9	156 (94.0%)	199 (99.0%)	0.007*
Systemic Anticoagulation Medication			1.0			0.02*
Apixaban	4 (1.3%)	7 (2.0%)		3 (1.9%)	4 (2.0%) 0	
Argatraban	0 (0%)	1 (0.3%)		1 (0.6%)	(0%)	
Dalteparin	16 (5.2%)	3 (0.8%)		1 (0.6%)	2 (1.0%)	
Edoxaban	1 (0.3%)	0 (0%)		0 (0%)	0 (0%)	
Enoxaparin	54 (18%)	106 (30%)		38 (24%)	68 (34%)	
Other	0 (0%)	1 (0.3%)		0 (0%)	1 (0.5%)	
Rivaroxaban	11 (3.6%)	10 (2.8%)		4 (2.5%)	6 (3.0%)	
Unfiltered Heparin	217 (71%)	228 (64%)		110 (70%)	118 (59%)	
Warfarin	2 (0.7%)	1 (0.3%)		1 (0.6%)	0 (0%)	
Time to Therapeutic AC (Hours)	8 (6, 20)	8 (6, 22)	0.6	8 (6, 23)	8 (6, 15)	0.3
Number of Procedures	66 (20.8%)	42 (11.4%)	0.01*	16 (9.5%)	26 (12.9%)	0.4
Procedural Intervention						
Catheter-Directed Lysis	15 (4.7%)	2 (0.5%)	< 0.001*	1 (0.6%)	1 (0.5%)	> 0.9
IVC Filter	42 (13.2)	24 (6.5%) 1	0.003*	13 (7.8%)	11 (5.5%) 1	0.4
Surgical Intervention	4 (1.3%)	(0.3%)	0.2	0 (0.0%)	(0.5%)	> 0.9
Thromboaspiration	5 (1.6%)	15 (4.1%)	0.050	2 (1.2%)	13 (6.5%)	0.015*
Systemic Thrombolysis	20 (6.3%)	19 (5.2%)	0.5	4 (2.4%)	15 (7.5%)	0.030*
Hospital Length of Stay (days)	6 (2, 11)	6 (3, 12)	0.12	9 (4, 18)	4 (2, 8)	< 0.001*
ICU Length of Stay (days)	0 (0, 4)	0 (0, 4)	0.6	2 (0, 7)	0 (0, 2)	< 0.001*
30-Day Mortality			0.78			0.0024*
Alive at 30 Days	276 (87.0%)	321 (87.0%)		132 (80%)	189 (94%)	
Dead at 30 Days	37 (12.0%)	43 (12.0%)		33 (20%)	10 (5.0%)	
Unknown	3 (1.9%)	3 (0.8%)		1 (0.6%)	2 (1.0%)	
Goals of Care Precluded PERT Consult	– –		–	12 (7.1%) N/A **–**		

^1^Median (IQR), *n* (%)

^2^Wilcoxon rank sum test, Pearson’s Chi-squared test, Fisher’s exact test

**Table 3:** Model-based estimates of 30-day mortality, hospital length-of-stay, time to therapeutic anticoagulation, and active bleeding, stratified by PERT presence and PERT consultation

	**PERT PRESENT**	**PERT CONSULTED**
**30 Day mortality**		
Odds ratio	1.06	0.34
95% Confidence Interval	0.70, 0.162	0.18, 0.61
* p*-value	0.8	< 0.001*
**Hospital length-of-stay**		
Beta	-0.19	-5.4
95% CI	-2.5, 2.1	-8.2, -2.5
* p*-value	0.9	< 0.001*
**Time to therapeutic anticoagulation**		
Odds ratio	0.15	-0.25
95% CI	-0.03, 0.33	-0.49, -0.01
* p*-value	0.10	0.041*
**Active bleeding**		
Odds ratio	0.99	0.28
95% CI	0.51, 1.90	0.09, 0.76
* p*-value	> 0.9	0.011*

### PERT Consultation

Three hundred sixty nine patients admitted after initiation of the PERT were included, of which 201 received PERT consultation. Age, sex, and PESI scores were similar between PC and NPC cohorts (Table 1). PC patients were more likely to have COPD, congestive heart failure, and history of thrombophilia and less likely to have cirrhosis or active cancer (p < 0.05). PCs were more likely to have complete risk stratification including both imaging and biomarker assessment of right ventricular strain[4] (OR 12.8, CI 6.6–24.7, *p* < 0.001), and were more likely to have RHS on imaging (*p* < 0.001). PCs had no difference in rates of catheter directed lysis (0.5% vs 0.6%, *p* > 0.9) or IVC filter (5.5% vs 7.8%, *p* = 0.4), but had statistically significantly higher rates of systemic thrombolysis (7.5% vs 2.4%, *p* = 0.030) and thromboaspiration (6.5% vs 1.2%, *p* = 0.015). PCs trended towards a higher number of total procedures (26 vs 16 procedures, *p* = 0.4). Controlling for basic demographics and common comorbidities, PC was associated with lower 30-day mortality (5.0% vs 20.0%, OR 0.38, CI 0.18–0.69, *p* = 0.0024), including 75% lower odds of 30-day mortality. Additionally, PC was associated with shorter HLOS (4 vs 9 days, -5.4 days, CI -8.2 – -2.5 days, *p* < 0.001), TAC (-0.25 h, CI -0.49 – -0.01 h, *p* = 0.041), and lower rates of in-hospital bleeding complications (OR 0.28, CI 0.09–0.76, *p* = 0.011).

## Discussion

Prior studies have detailed single-center experiences of PERT implementation with heterogenous results, often suggesting that hospital adoption of PERT could improve patient outcomes by rapidly identifying intermediate and high-risk cases and increasing institutional knowledge of PE evaluation and management, regardless of PERT consultation [3, 13, 14]. Though few have assessed the direct impact of PERT consultation on clinical outcomes and hospital utilization metrics.

### Clinical outcomes

Consistent with prior studies, we found no significant difference in 30-day mortality between the pre- and post-PERT cohorts [15–20]. However, when assessing the impact of PERT consultation, there was a markedly reduced 30-day mortality for the PC cohort, despite PCs having similar PESI scores. Furthermore, PCs were more likely to have intermediate-high and high-risk PEs when compared to NPCs (*p*< 0.004). PC patients had a slightly lower 30-day mortality than the 6.5% reported by The PERT Consortium™ for PC patients with intermediaterisk PE [21].Of the two prior studies comparing PC to NPC cohorts, neither demonstrated a significant difference in mortality [18, 20]. Notably, these studies included patients who were admitted with low-risk PE, which may have obscured any potential benefit for higher risk populations.

We propose several factors that may explain the difference in mortality seen in our study. PC patients were much more likely to receive a complete guideline-directed PE risk stratification evaluation. This, coupled with reduced TAC, reduced in-hospital bleeding complications, and fewer procedures may suggest a more effective evaluation of patient-specific factors and therapy-selection following the multidisciplinary PERT discussion.

Reduced TAC, as seen in the PC cohort may play an important role. Professional guidelines recommend prompt initiation of anticoagulation in acute PE, even prior to diagnostic confirmation when there is high clinical suspicion [4]. Prior research has shown an increase in mortality for every hour delay in diagnosis, and that patients who achieve therapeutic anticoagulation within 24 h have a reduced in-hospital and 30-day mortality [22, 23]. This suggests a time-sensitive benefit to reperfusion. The noted reduction in TAC may be partially driven by the PC cohort being more likely to receive enoxaparin as the initial anticoagulant,

which frequently achieves therapeutic levels more rapidly than heparin infusions. Notably, patients in the PC cohort were less likely to have in-hospital bleeding complications despite being more likely to be initiated on enoxaparin. A potential confounder is differences in baseline comorbidities between PCs and NPCs, with PCs being more likely to have COPD, congestive heart failure, and thrombophilia, but less likely to have active cancer and cirrhosis. Despite higher rates of active cancer, NPC patients rarely had documented goals of care that would preclude PERT consultation or consideration of procedures, which may suggest perceived futility of consultation in this population.

### Interventions

When comparing the pre- vs post-PERT eras, our study demonstrated no significant difference in the outcomes of interest noted above; however, there were significantly fewer procedures in the post-PERT era. This was driven largely by a statistically significant reduction in catheter-based procedures as well as decreased IVC filter placement. There was also a non-significant trend towards a concomitant increase in thromboaspiration procedures. There were no institutional changes in the types of available therapies during our study period. Notably, there was no difference in rates of systemic lysis in the pre- vs post-PERT eras. In contrast, most studies including a recent meta-analysis demonstrate an overall increase in advanced therapies following PERT implementation, which is typically defined as both procedural interventions and.

systemic lysis.10,17,19–23.

The reduction in procedures observed following initiation of PERT at our center stands in.

contrast to prior studies that reported an increase in procedures following PERT initiation [6, 20, 23, 24]. Importantly, this reduction in procedures was not associated with a significant difference in mortality, HLOS, or TAC. There are several factors likely contributing to the reduction in procedures noted in our study. For one, this difference may be due our institution’s “gatekeeper” PERT model: PERT consults are initially evaluated by a pulmonologist on the Pulmonary Hypertension Service prior to involvement of interventional specialties. In this “gatekeeper” model, the pulmonologist determines whether involvement of other services is warranted, and the decision regarding advanced therapies is then made with the full multidisciplinary PERT. As a result, interventional specialties are involved in a minority of cases. Not all PERTs are structured in this way, with some centers activating all PERT services simultaneously for a coordinated multidisciplinary evaluation or have non-pulmonary services in the gatekeeper role. This heterogeneity of PERTs at different institutions may limit the generalizability of these results to other centers. Secondly, thromboaspiration has been an increasingly appealing option compared to CDT due to decreased need for ICU monitoring and concerns for non-trivial bleeding risk associated with CDT. PC patients were more likely to undergo thromboaspiration or receive systemic thrombolysis than NPC patients, while having reduced rates of bleeding during hospitalization. This may suggest more effective therapy selection following the multidisciplinary discussion with PERT involvement. The significant reduction in IVC filter placement likely reflects a change in global practice pattern after large randomized-controlled trials, published after the first PERT was established, showing no significant benefit in routine IVC filter placement in select populations [24, 25]. This is supported by the nearly ubiquitous demonstration of reduced IVC filter use in studies comparing pre- vs post-PERT eras, including a recent meta-analysis by Sosa et al. [9, 10, 15] As further evidence, the rates of IVC filter placement did not vary amongst PC and NPC cohorts in the PERT era.

Importantly, this two-tiered analysis highlights possible confounding factors present in prior prepost PERT studies. The fact that PERT consultation, rather than PERT presence, was associated with significant benefit to patients, suggests that PERT consultation may have previously unappreciated benefits. Studies of the effect of PERTs with a pre-post design may be confounded by global practice changes, non-consultation, or lack of involvement by the PERT.

### Limitations

There are several limitations to our study, including the retrospective nature and single-center design Despite the authors attempts to address potential confounding with the use of propensity score matching, PC patients and NPC patients may have intangible differences that we were not able to capture through review of the medical records. Additionally, our extensive institutional experience with interventions such as thromboaspiration and catheter-based therapies, and comparatively limited experience ECMO, may limit the generalizability of our conclusions to centers with similar experiences and resources. Furthermore, the significant heterogeneity of PERTs in terms of PE severity, frequency of activation, interventions available, and team composition may explain the subsequent heterogeneity in reported outcomes [10]. Thus, our results may only be generalizable to institutions with similar PERT composition and resources.

## Conclusions

In summary, we observed a significant reduction in 30-day mortality, TAC, HLOS, and inhospital bleeding complications for patients who received PERT consultation. These differences were not observed when evaluating the impact of PERT presence in pre-vs post-implementation eras. Additionally, there was no observed increase in the use of advanced therapies in the postimplementation era. Together, this suggests that consultation of multidisciplinary PERT, rather than the mere existence of PERT at our institution, may have previously unappreciated benefits to select patients with acute intermediate or high-risk PE, and can be achieved without a concomitant increase in advanced therapies. Given the single-center retrospective nature of our study and the large treatment effect observed, these results must be interpreted with caution. Importantly, this two-tiered analysis highlights possible confounding factors present in prior prepost PERT studies with a pre-post design, which may be confounded by global practice changes, non-consultation, or lack of involvement by the PERT. Further investigation is needed to explore the impact of PERT composition on various outcomes, barriers to PERT consultation, and long-term effects of PERT involvement.

### Supplementary Information

**Supplementary Material 1.**

## Data Availability

No datasets were generated or analysed during the current study.
