# Supplementary material for: Reduced mortality associated with pulmonary embolism response team consultation for intermediate and high-risk pulmonary embolism: a retrospective cohort study
Source: Thromb J. 2024 Apr 19;22:38. doi: 10.1186/s12959-024-00605-8 (PMC11027408; doi:10.1186/s12959-024-00605-8)
Supplement: Supplementary file 1 — Supplementary Material 1. [file 12959_2024_605_MOESM1_ESM.docx]

PERT Followup Appendix

Appendix 1:

**Chart Review:**

*Chart Reviewer Training:*

Data elements for chart review were determined by collaborative decision making by all coauthors. Data extraction was performed by two internal medicine residents familiar with medical language, both of whom had conducted previous observational studies. Ten example cases were completed as part of a training procedure to standardize the workflow for data extraction and entry into a REDCap database.^1^

*Case Selection:*

Inclusion criteria included age >18 and inpatient admission to the University of Colorado Hospital System with concomitant diagnosis of intermediate or high-risk pulmonary embolism.

*Data Abstraction Form and Definition of variables*

The research team used a REDCap survey form to extract and enter data. The codebook for data extraction including definitions of variables can be reviewed in a supplemental table.

*Inter-rater reliability for chart review:*

A randomly selected subset of 10 variables from 10 randomly selected charts underwent analysis for interrater reliability, with data entry being completed by two research team members. Cohen’s kappa statistic was calculated for each variable.


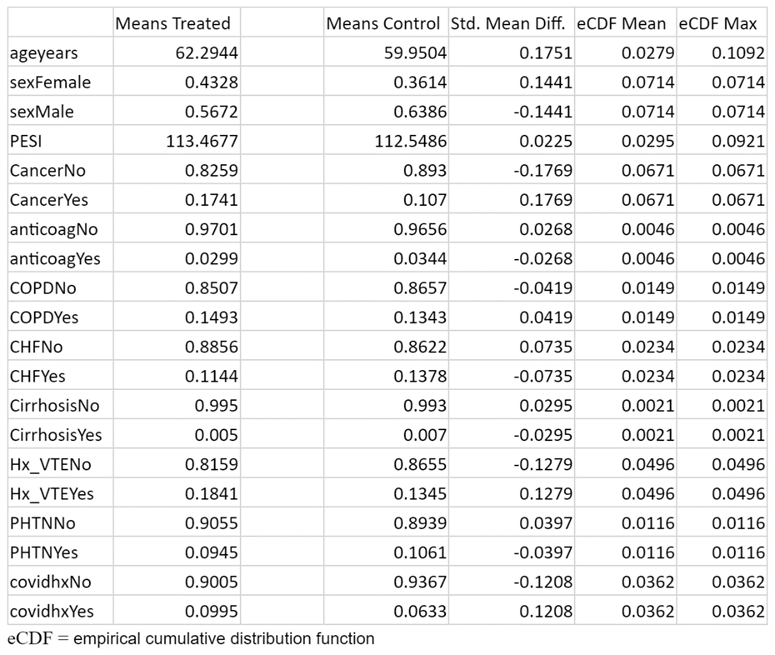


| Variable | Kappa-statistic | p-value |
| --- | --- | --- |
| Home Use of Systemic Anticoagulation | 1 | 0.002 |
| Active Bleed during Index Hospitalization | 1 | <0.001 |
| Echocardiographic Evidence of Right Heart Strain at time of PE Diagnosis | 0.75 | 0.03 |
| DVT Ultrasound Performed during Index Hospitalization | 1 | 0.002 |
| Follow-up Echocardiogram Completed | 0.74 | 0.02 |
| IVC Filter Placed | 1 | 0.002 |
| Initial Troponin at time of PE diagnosis | 0.88 | <0.001 |
| Initial NT-ProBNP at time of PE Diagnosis | 1 | <0-.001 |
| Discharged on Systemic Anticoagulation | 1 | 0.003 |

Table 1: Kappa statistics for inter-rater reliability assessment of data extracted during chart review. Values above 0.6 suggest moderate agreement, above 0.8 strong agreement, and 0.9 near-perfect agreement. PE = Pulmonary Embolism, DVT = Deep Venous Thrombosis, IVC = Inferior Vena Cava, NT-ProBNP =N-terminal Pro-Brain Natriuretic Peptide

Appendix 2:


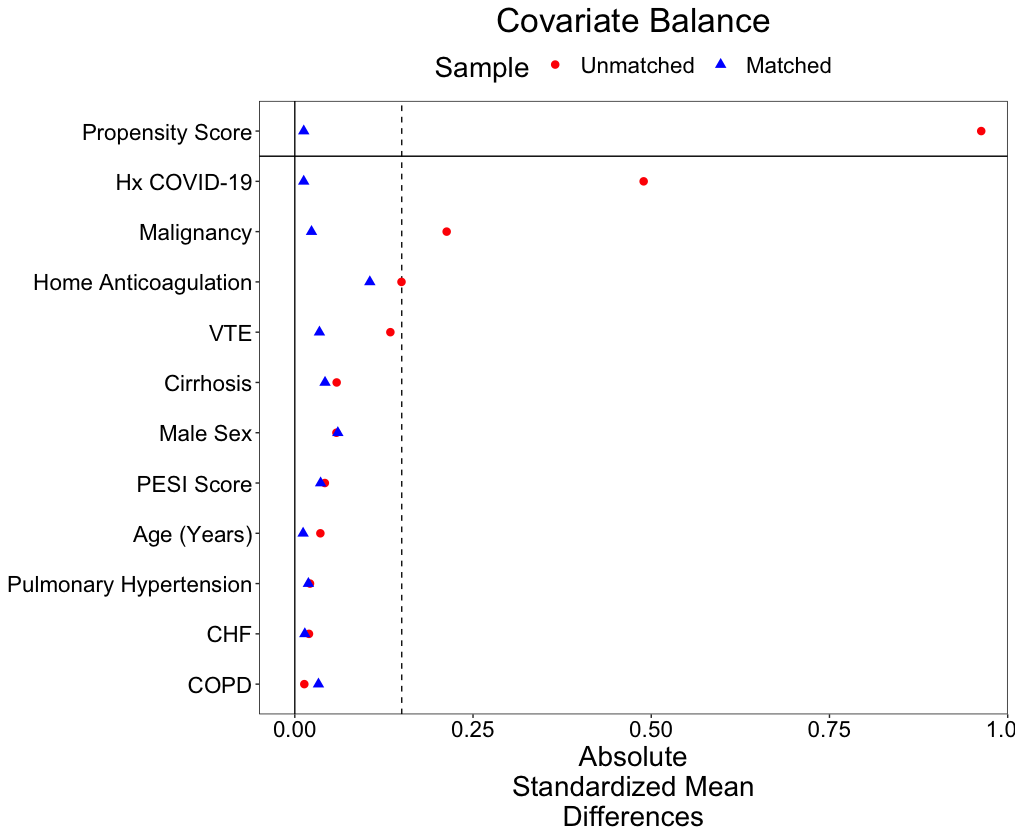


Figure 1: Love plot showing absolute standardized mean differences of covariates between matched and unmatched patients before and after PERT initiation. Difference in Absolute Standardize Mean Differences Below 0.2 suggest an acceptable degree of matching


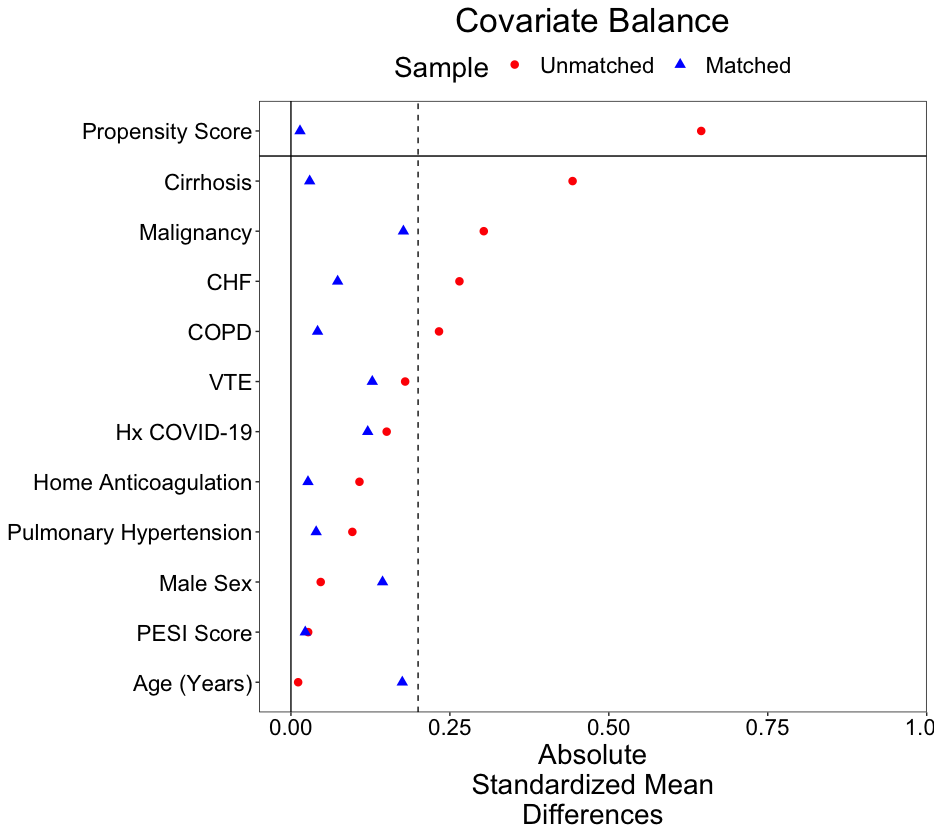


Figure 2: Love Plot of Standardized Mean Differences of covariates before and after full matching. Difference in Absolute Standardize Mean Differences Below 0.2 suggest an acceptable degree of matching.

**Appendix 3:**

PERT Establishment:

The Pulmonary Embolism Response Team (PERT) at the University of Colorado Hospital (UCH) was initiated on 4/1/2019. PERT structure was determined through a multi-disciplinary consensus process including representatives from pulmonology, cardiothoracic surgery, and interventional and diagnostic radiology. After a run-in period during which all services were educated about the development and availability of the PERT, the electronic health record (EHR) was updated to reflect pathways and consult options for the PERT.

PERT Process:

At the time that a provider has a suspicion for intermediate or high-risk PE, it is recommended that they consult the PERT through the EHR and by pager. Each PERT consult is evaluated by an attending pulmonologist with subspecialty training in pulmonary vascular disease. After initial evaluation, the PERT consultant decides whether to activate a larger team including providers from the interventional radiology and cardiothoracic surgery services to discuss possible interventions.

PERT Follow-up:

Patients are referred to a dedicated PE follow-up clinic in the three months after discharge. This follow-up includes assessment of functional status, discussion of anticoagulation strategy, and if appropriate, further evaluation and diagnostic workup for chronic thromboempolic pulmonary hypertension.
